# Genetic Background Predicts Uveal Melanoma Patients’ Outcomes

**DOI:** 10.1016/j.xops.2025.100972

**Published:** 2025-10-10

**Authors:** Thibault Verrier, Anaïs Le Ven, Alexandre Houy, Erwin Brosens, Emine Kilic, Wishal D. Ramdas, Tolga Bicer, Agathe Garcia, Anne-Charlotte Lefranc, Sandra Vanhuele, Amanda F. Kahn, Gaelle Pierron, Alexandre Matet, Nathalie Cassoux, Chrystelle Colas, Manuel Rodrigues, Josselin Noirel, Marc-Henri Stern

**Affiliations:** 1 INSERM U1339 CNRS UMR3666, DNA Repair and Uveal Melanoma (D.R.U.M.), Institut Curie, Paris, France; 2 Université Paris Sciences & Lettres, Paris, France; 3 Département de génétique, Institut Curie, Paris, France; 4 Clinical Genetics Department, Erasmus MC, Rotterdam, The Netherlands; 5 Erasmus MC Cancer Institute, Erasmus MC, Rotterdam, The Netherlands; 6 Department of Ophthalmology, Erasmus MC, Rotterdam, The Netherlands; 7 Département d’Oncologie Oculaire, Institut Curie, Paris, France; 8 Université Paris Cité, Paris, France; 9 Département d’Oncologie médicale, Institut Curie, Paris, France; 10 Laboratoire GBCM (EA7528), Conservatoire National des Arts et Métiers (CNAM), Paris, France

**Keywords:** Genetic background, Predictive model, Prognostication, Uveal melanoma

## Abstract

**Objective:**

Single nucleotide polymorphisms (SNPs) in *IRF4* and *HERC2* are associated with risk for disomy or monosomy of chromosome 3 (D3 or M3) uveal melanoma (UM), respectively. The aim of this study was to assess the association between germline genetics and UM outcome and the potential use of a derived prognostic signature for UM.

**Design:**

Cohort study from Institut Curie, Paris (France) and Erasmus University Medical Center, Rotterdam (The Netherlands).

**Participants:**

Patients diagnosed with UM at Institut Curie (*N* = 2059) and Erasmus University Medical Center (*N* =576).

**Methods:**

Impact of *IRF4* and *HERC2* SNPs on survival was assessed in a cohort of 1339 patients with UM by Kaplan-Meier analysis and Cox proportional hazard regression. Uveal melanoma subtype-specific risk associations with SNPs and iris color were assessed by generalized linear model regression analyses. Classifier of UM subtypes was trained on 560 patients with UM and validated in 2 independent cohorts.

**Main Outcome Measures:**

We analyzed risk SNPs in the series of patients with UM in relation to tumor and patient characteristics, including eye color, tumor subtype and diameter, and patient outcomes.

**Results:**

*IRF4* rs12203592-T and *HERC2* rs12913832-G SNPs were associated with improved and worsened progression-free-survival and overall survival, respectively, mainly through their association with chromosome 3 status. Associations between *IRF4* and *HERC2* risk SNPs and D3 or M3 subtypes, respectively, were largely independent of their role in determining iris pigmentation. A genetic classifier showed significant results in predicting chromosome 3 status and survival but did not outperform established clinical prognostic features.

**Conclusions:**

Our study demonstrates that inherited polymorphisms in *IRF4* and *HERC2* are independently associated with UM subtype and prognosis, although a SNP-based classifier does not yet outperform the established prognostic model.

**Financial Disclosure(s):**

Proprietary or commercial disclosure may be found in the Footnotes and Disclosures at the end of this article.

Uveal melanoma (UM) is the most frequent intraocular primary tumor in adults. Uveal melanoma arises from the uveal tract, mainly from the choroid (>90%), and more rarely from iris and ciliary body.^1^ Although primary UMs are generally efficiently treated, metastatic UM arises in nearly half of UMs and is associated with a grim prognosis because of few effective treatments, with a median survival time <14 months and a remarkable hepatic tropism.^1^ Different UM subtypes have been defined either by gene expression profiling (GEP): GEP class 1 and class 2; by chromosome 3 genomic status: monosomy 3 (M3) and disomy 3 (D3); or by multiomics approaches generating 4 clusters, 1 to 4.^2^, ^3^, ^4^ These UM subtypes differ greatly in their biology and prognosis. GEP class 1/D3/clusters 1 and 2 UM are associated with fair to good prognosis and with mutually exclusive mutations in *SF3B1* and *EIF1AX*, which encode a key splicing factor and a translation initiation factor, respectively.^4^ Conversely, GEP class 2/M3/clusters 3 and 4 UM are associated with high metastatic risk, poor prognosis, and inactivating mutations in *BAP1*, which encodes a ubiquitin carboxy-terminal hydrolase.^4^ Uveal melanoma prognostication thus relies on genetic and molecular characterization, which requires tumor samples, generally obtained by fine-needle aspiration biopsy or after enucleation. Although fine-needle aspiration biopsy is regarded as safe, it is still an invasive procedure with potential ocular complications, and its use differs according to medical practice.^5^, ^6^, ^7^, ^8^ Uveal melanoma biopsy in Europe is not systematically performed^5^ and is restricted to large tumors; thus, developing markers of prognostication that do not require biopsy is of prime importance for clinicians treating UM.

Uveal melanoma has a singular epidemiology and mainly affects individuals of European ancestry, with a 10- to 20-fold greater incidence than in individuals of non-European ancestry.^9^ Fair skin and light eye color are also known UM risk factors.^10^^,^^11^ Despite the similarities with cutaneous melanoma, UM is a very different cancer that does not exhibit any ultraviolet mutational signature, except for iris melanomas.^12^, ^13^, ^14^

We previously published large UM genome-wide association studies (GWASs) and identified 3 loci associated with UM risk: *TERT/CLPTM1L*, *IRF4*, and *HERC2*, with leading single nucleotide polymorphisms (SNPs) rs421284, rs12203592, and rs12913832, respectively,^15^^,^^16^ which were also suggested in a candidate association approach.^17^ *IRF4* and *HERC2* SNPs are 2 major variants influencing eye pigmentation.^18^^,^^19^ Strikingly, *HERC2* rs12913832 is solely associated with a risk for M3 UM, whereas *IRF4* rs12203592 is solely associated with a risk for D3 UM.^16^ A recent meta-analysis of UM GWASs fully confirmed our results.^20^ Interestingly, Gelmi et al^21^ from the group of M.J. Jager showed the association of *HERC2* rs12913832-G with M3 status and UM survival, without identifying the opposite association with *IRF4* rs12203592.

Here, we confirmed the association between *HERC2* rs12913832 and *IRF4* rs12203592 with UM chromosome 3 status. We showed the association of *HERC2* and *IRF4* SNPs with survival of patients with UM, through their contribution to the M3 and D3 UM risk, and independent of their role in eye pigmentation. Finally, we assessed the predictive value of these SNPs to determine chromosome 3 status and UM outcome, as a potentially noninvasive prognostication of UM.

## Methods

### Samples from Patients with UM and Control Individuals

The study was performed in accordance with the ethical principles of the Declaration of Helsinki and approved by the ethical committee and internal review board at the Institut Curie (DATA230128). Blood samples were obtained from 2059 patients with UM who consented to participate in the study and from 925 control individuals of French origin from the KIDRISK consortium (US NCI U01CA155309; G. Scelo).^16^ Compared with the previously reported GWAS,^16^ an additional series of 465 UM were genotyped on the Infinium Global Screening Array 24 v1.0 (Illumina) at the *Centre National de Génotypage* (Evry, France) from patients’ blood DNA. Genotypes of 576 UM cases—the Rotterdam Ocular Melanoma Study (ROMS) cohort—were obtained from Erasmus University Medical Center, Rotterdam, The Netherlands, genotyped on the Infinium Global Screening Array 24 v1.0 and used as a validation set. The Medical ethics committee of the Erasmus Medical Centre (OZR nr 2009-17, MEC-2009-375, November 12, 2009) approved the study. Clinical and genetic features of each cohort are presented in the supplementary materials.

### Genotyping, Imputation, and Merge

Genotyped samples from previous GWASs, 511 new UM cases, and control cohorts were independently filtered and imputed on the TOPMed Imputation Server using Eagle for phasing and the TOPMed Imputation Panel version r3 as the reference data set.^16^^,^^22^^,^^23^ Individuals and markers were filtered based on call rate (>95%), minor allele frequency (>1%), and concordance between genetic and reported sex, as well as relatedness. Imputed data sets were merged and then went through another quality control that included selection of samples of European ancestry and outlier filtering by principal component analysis using the smartPCA software,^24^ as previously described.^16^

Eye color was assessed by the ophthalmologists. When an ambiguous color was noted (hazel, green-brown, and blue-brown), IrisPlex was used to further determine eye color as brown, green, or blue.^25^ The ROMS cohort was subjected to the same quality control and imputation pipelines, and eye color annotation was predicted using IrisPlex.

### UM Tumor Samples

DNA from UM tumor samples from therapeutic enucleation was used to determine chromosome 3 status, as well as *EIF1AX* and *SF3B1* mutational status, as part of routine diagnostic testing.

### Statistical Analysis

Statistical analyses were performed using R v4.4.1. Association tests were performed using the “glm” function from the stats package with the logit link function. Each regression was adjusted for patient age at diagnosis and sex as covariates. Associations were determined by odds ratios (ORs) with 95% confidence intervals (CIs), with a *P* value of ≤0.05 considered significant. Survival analyses were performed with Kaplan-Meier curves with log-rank tests and Cox proportion hazard regression. Predictive models were built and trained with the ‘glm’ function and compared with likelihood ratio tests with the ‘lrtest’ function from the ‘lmtest’ package. Progression-free survival (PFS) events were defined as the presence of metastases or death for the Curie series. This information was not available for the ROMS series. Overall survival (OS) events were defined as UM-related death for the ROMS series. However, the precise cause of death for the retrospective Curie series was not available, and OS events were defined as all-cause death, as recommended.^26^

For model training and testing, samples from 560 patients of UM with known chromosome 3 status from Institut Curie series were used as the training set. Two validation series were constituted of the following: (1) samples from 239 patients with UM with known chromosome 3 status from Institut Curie, with no overlap with the training set (validation series #1), and (2) samples from 196 UM patients with available chromosome 3 status from the ROMS cohort (validation series #2).

Linkage disequilibrium between the *HERC2* rs12913832 and *IRF4* rs12203592 UM risk SNPs was computed based on the 1000G phase 3 data (non-Finnish European subset, *N* = 404, GRCh38 build) using the web-based application LDlink using the “LDpairs” tool available at https://ldlink.nih.gov.^27^

## Results

### UM Molecular Subtypes and Eye Pigmentation

**Figure 1:**
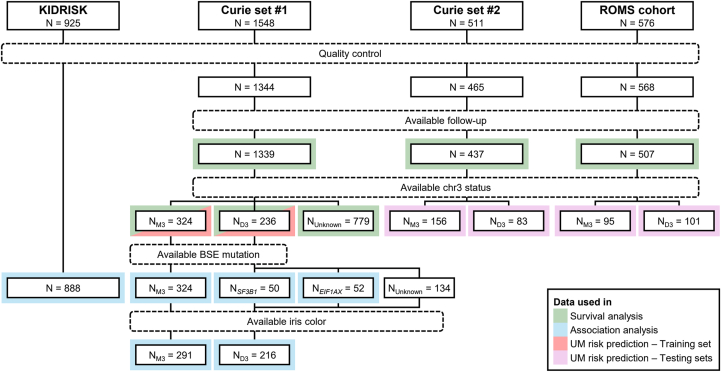
Flowchart of the study. Square-edged boxes show the number samples at each step. Round-edged and hatched boxes represent filtering steps. Gray boxes represent the different analyses. D3 = disomy of chromosome 3; M3 = monosomy of chromosome 3; UM = uveal melanoma.

The *HERC2* rs12913832 and *IRF4* rs12203592 UM risk SNPs, preferentially associated with M3 and D3 UM, respectively, are known to play a major role in eye pigmentation.^16^^,^^18^^,^^19^ Notably, *IRF4* and *HERC2* are located on chromosomes 6 and 15, respectively, and thus the distribution of their genotypes is independent (*R*^2^ = 0.002, *P* = 0.2). We assessed whether the UM risk carried by the *HERC2* and *IRF4* SNPs was linked to their role in eye pigmentation. We analyzed a series of 1339 UM cases of European ancestry with qualified genotyping and annotated follow-up (Fig 1; Table 1).

**Table 1:** Characteristics of the Series

Chromosome 3 Status *N*	Curie Set #1			Curie Set #2			ROMS set			KIDRISK Control Set
	D3	M3	Unknown	D3	M3	Unknown	D3	M3	Unknown	Controls
	236	324	809	83	156	206	95	101	335	888
**Individual characteristics**										
Sex										
Male	135 (57%)	158 (49%)	364 (46%)	45 (54%)	74 (47%)	103 (52%)	61 (55%)	46 (44%)	167 (50%)	658 (74%)
Female	101 (43%)	166 (51%)	419 (54%)	38 (46%)	82 (53%)	95 (48%)	49 (45%)	58 (56%)	170 (50%)	230 (26%)
Age at diagnosis, y	58 (14)	62 (13)	63 (14)	63 (14)	67 (12)	61 (15)	62 (12)	64 (13)	62 (13)	62 (11)
Eye color∗										
Brown	95 (44%)	104 (36%)	255 (34%)	28 (37%)	37 (28%)	53 (31%)	35 (32%)	20 (19%)	65 (19%)	NA
Green	29 (13%)	21 (7.2%)	88 (12%)	9 (12%)	12 (9.2%)	28 (16%)	2 (1.8%)	0 (0%)	1 (0.3%)	NA
Blue	92 (43%)	167 (57%)	401 (54%)	39 (51%)	81 (62%)	90 (53%)	73 (66%)	84 (81%)	271 (80%)	NA
Unknown	20	33	39	7	26	27				
**Tumor characteristics**†										
Diameter	15.0 (3.6)	16.4 (3.6)	11.1 (3.4)	17.9 (3.2)	17.9 (4.1)	11.2 (3.5)	12.5 (3.6)	13.9 (4.0)	11.9 (3.4)	NA (NA)
Thickness	9.4 (2.9)	9.6 (3.2)	4.9 (2.4)	10.9 (2.5)	10.9 (3.3)	4.6 (2.2)	9.4 (23.9)	8.0 (3.7)	5.9 (3.1)	NA (NA)
**Genetics**										
*CLPTM1L*										
T/T	58 (25%)	77 (24%)	170 (22%)	22 (27%)	23 (15%)	60 (30%)	30 (27%)	25 (24%)	77 (23%)	260 (29%)
C/T	106 (45%)	162 (50%)	396 (51%)	41 (49%)	87 (56%)	89 (45%)	48 (44%)	49 (47%)	169 (50%)	464 (52%)
C/C	72 (31%)	86 (26%)	217 (28%)	20 (24%)	46 (29%)	49 (25%)	32 (29%)	30 (29%)	91 (27%)	164 (18%)
*IRF4*										
C/C	118 (50%)	226 (70%)	435 (56%)	48 (58%)	111 (71%)	103 (52%)	79 (72%)	87 (84%)	258 (77%)	643 (72%)
C/T	99 (42%)	92 (28%)	312 (40%)	31 (37%)	45 (29%)	84 (42%)	29 (26%)	17 (16%)	78 (23%)	227 (26%)
T/T	19 (8.1%)	7 (2.2%)	36 (4.6%)	4 (4.8%)	0 (0%)	11 (5.6%)	2 (1.8%)	0 (0%)	1 (0.3%)	18 (2.0%)
*HERC2*										
A/A	31 (13%)	25 (7.7%)	98 (13%)	11 (13%)	10 (6.4%)	22 (11%)	4 (3.6%)	0 (0%)	3 (0.9%)	156 (18%)
A/G	114 (48%)	113 (35%)	279 (36%)	33 (40%)	56 (36%)	75 (38%)	31 (28%)	20 (19%)	62 (18%)	424 (48%)
G/G	91 (39%)	187 (58%)	406 (52%)	39 (47%)	90 (58%)	101 (51%)	75 (68%)	84 (81%)	272 (81%)	308 (35%)

D3 = disomy of chromosome 3; M3 = monosomy of chromosome 3; NA = not applicable.

Data shown as n (%) or mean (SD).

∗ Eye color was observed by the ophthalmologist for the Curie series, and predicted using the IrisPlex algorithm for the ROMS uveal melanoma cohort.

† As measured by B-mode ultrasonography.

**Figure 2:**
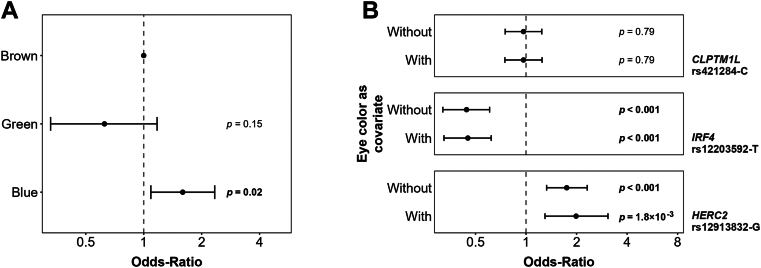
Effects of eye color on the uveal melanoma (UM) risk. Odds ratios (ORs) obtained with logistic regression comparing 291 monosomy 3 (M3) to 216 disomy 3 (D3) UMs, adjusted for patients’ age at diagnosis and sex as covariates. **A,** Sole effect of eye pigmentation on M3/D3 risk. Odds ratio corresponds to the risk of developing a M3 UM compared with D3 UM. **B**, Effect of the 3 loci associated with UM risk (*CLPTM1L*, *IRF4*, and *HERC2*) on M3/D3 risk with or without eye color as covariate. The error bars represent the 95% confidence intervals of the ORs. Statistical significance is assessed by a 2-tailed Z-score test. The vertical dotted line is set at OR = 1.00, indicating an absence of association with M3/D3 risk.

First, we evaluated the effect of observed eye colors in a series of patients with UM with available chromosome 3 status and iris color information (*n* = 507; M3, *n* = 291 and D3, *n* = 216) by logistic regression analysis on M3/D3 risk after adjustment for age, sex, and population structure. Blue eye color was associated with an OR of 1.59 (95% CI, 1.09–2.34) for M3 risk (Fig 2A; Table S2, available at https://www.ophthalmologyscience.org), and eye color was found significantly associated with the chromosome 3 status by likelihood ratio test (*P* = 0.003).

Second, we wished to evaluate whether the UM risk SNPs confer their risk through their role on eye pigmentation. Therefore, we applied the conditional analysis approach that excludes the influence of a covariate (in this case, eye color) from the measured association. We evaluated the association of 3 UM risk alleles (*CLPTM1L* rs421284-C, *IRF4* rs12203592-T, and *HERC2* rs12913832-G) with chromosome 3 status, including or not including eye color as a covariate in the logistic regression model on M3/D3 risk. The inclusion of the eye color covariate did not modify the M3/D3 OR for these 3 risk SNPs (Fig 2B; Table S3, available at https://www.ophthalmologyscience.org). We confirmed that *CLPTM1L* rs421284-C was not associated with chromosome 3 status with or without actual eye color as a covariate (Table S3), whereas *HERC2* and *IRF4* were both significantly associated with chromosome 3 status, without significant changes in OR. This conditional analysis strongly supported that the association of *HERC2* and *IRF4* with M3/D3 risk is independent of their role in eye pigmentation.

### *IRF4* Polymorphism is Associated with Both *SF3B1*- and *EIF1AX-*mutated D3 UM

**Figure 3:**
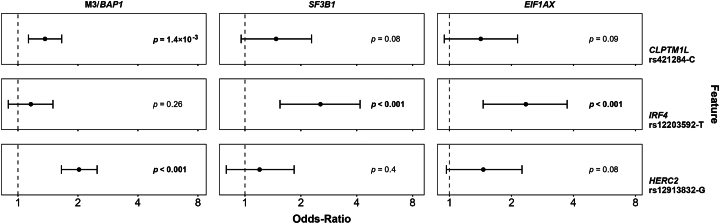
Genetic risk factors for *SF3B1-* and *EIF1AX-*mutated uveal melanoma (UM). Odds ratios (ORs) obtained by logistic regression (generalized linear model) comparing 324 *BAP1-*mutated, 50 *SF3B1-*mutated, or 52 *EIF1AX-*mutated UMs to 888 controls and adjusted for patient age at diagnosis and sex as covariates. Each panel shows the risk effect of the 3 loci (*CLPTM1L*, *IRF4*, and *HERC2*) associated with each UM subtype. The error bars represent the 95% confidence interval of the OR. Statistical significance is assessed by a two-tailed Z-score test. The vertical dotted line is set at OR = 1.00.

We then wondered if the D3 UM risk allele *IRF4* rs12203592-T was preferentially associated with either *SF3B1*- or *EIF1AX-*mutated cases. We further extracted, from the 1339 samples, the mutation status of *SF3B1* and *EIF1AX* when the chromosome 3 status is known to be diploid (*n* = 236). The 50 *SF3B1* and 52 *EIF1AX* mutated samples were used to assess the association of the 3 UM risk SNPs versus a series of 888 healthy controls, after adjustment for patient sex, age at diagnosis, and population structure. We used a series of 324 patients with *BAP1*-mutated and/or M3 status as negative and positive controls for association with *IRF4* an*d HERC2* SNPs, respectively. This analysis showed that the *IRF4* SNP was significantly associated with both *SF3B1*- and *EIF1AX-*mutated D3 UMs (Fig 3; Table S4, available at https://www.ophthalmologyscience.org). As expected, *HERC2* was not associated with either subtype of D3 UM. The absence of association of *CLPTM1L* with both *SF3B1* and *EIF1AX*-mutated D3 UMs was probably due to the low number of samples.

### *HERC2* and *IRF4* Germline Risk Alleles Are Associated with Outcome of Patients with UM

**Figure 4:**
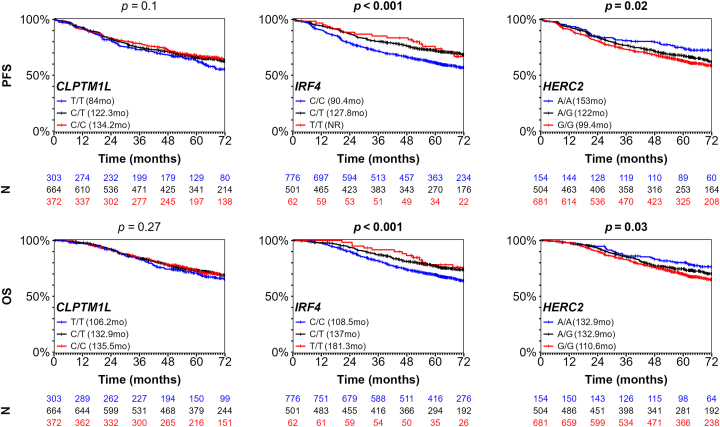
Kaplan-Meier (KM) analyses of progression-free survival (PFS) and overall survival (OS) according to genotypes. KM plots associated with PFS (top panel) and OS (bottom panel) of 1339 patients with UM stratified by genotype of rs421284 (*CLPTM1L*) (left panel), rs12203592 (*IRF4*) (middle panel), and rs12913832 (*HERC2*) (right panel). Median time in months is shown in parenthesis (NR = median not reached). *P* values were obtained from log-rank test with genotypes coded as 0, 1, or 2 according to the number of risk alleles carried. The numbers of individuals for each KM analysis and for all genotypes are shown below the KM graph. Censored individuals are noted with a vertical bar.

Given that genotypes of *HERC2* and *IRF4* are specifically associated with risk for M3 and D3 UM, respectively, and that loss of chromosome 3 is strongly associated with metastatic progression,^3^^,^^4^ we assessed whether *HERC2* rs12913832-G and *IRF4* rs12203592-T alleles could be associated with UM patient PFS and overall survival (OS). We analyzed the series of 1339 UMs and stratified according to the tested SNP genotypes (Fig 1). Kaplan-Meier survival analyses were performed for *HERC2* rs12913832-G, *IRF4* rs12203592-T, and *CLPTM1L* rs421284-C to assess their effects on PFS and OS. Stratification by *IRF4* genotype showed a better PFS, OS, and mean survival time for homozygous or heterozygous risk allele (T/T and C/T) carriers than for homozygous protective allele (C/C) carriers (Fig 4; Table S5, available at https://www.ophthalmologyscience.org). Conversely, stratification by *HERC2* genotype showed a poor, intermediate, and good PFS and OS for homozygous risk allele (G/G), heterozygous risk allele (A/G) and homozygous protective allele (A/A) carriers, respectively (Fig 4; Table S5). Stratification by *CLPTM1L* genotype showed no significant difference in PFS or OS time across the 3 genotypes (Fig 4; Table S5). We also confirmed the association of PFS and OS with *IRF4* and *HERC2* genotypes by univariate and multivariate Cox proportional hazard regression analyses (Tables S6 and S7, available at https://www.ophthalmologyscience.org). In summary, these specific germline risk alleles correlate with patient outcomes, with good prognosis/D3 associated with *IRF4* rs12203592-T, poor prognosis/M3 associated with *HERC2* rs12913832-G, and no effect of *CLPTM1L* rs421284-C.

To exclude a confounding association of tested alleles with a general effect on survival unrelated with UM, we performed the same analyses after stratification of cases according to their chromosome 3 status (M3, *n* = 324; D3, *n* =236). Separate Kaplan-Meier survival analyses performed within the M3 and D3 UM groups showed no significant difference in PFS and OS when stratified by their risk SNP genotypes (Figs S5 and S6, available at https://www.ophthalmologyscience.org). These findings were confirmed by Cox proportional hazards model multivariate analyses on PFS and OS, including the 3 SNP genotypes and chromosome 3 status (Table S8, available at https://www.ophthalmologyscience.org). These conditional analyses demonstrated that *HERC2* and *IRF4* are associated with PFS and OS, mainly through their association with chromosome 3 monosomy/disomy status.

### Building a Predictive Outcome Model Based on Genetic Background

To assess whether *HERC2* rs12913832-G and *IRF4* rs12203592-T could be used to predict UM chromosome 3 status, we used the same series of patients with UM of known chromosome 3 status to test 3 statistical models (Fig 1). Using univariate logistic regression, both SNPs (*IRF4* and *HERC2*), age at diagnosis and primary tumor largest basal diameter (LBD) were found to be significantly associated with chromosome 3 status. Tumor thickness and sex were not significantly associated (*P* = 0.42 and *P* = 0.06) with chromosome 3 status and were thus removed from subsequent analyses (Table S9, available at https://www.ophthalmologyscience.org).

**Figure 7:**
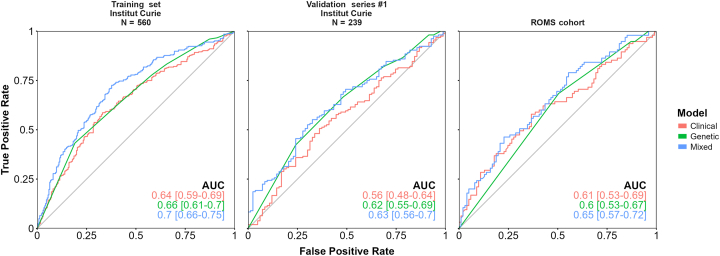
Predictive models of uveal melanoma (UM) risk. Receiver operating characteristic (ROC) curves and area under the curve (AUC) of training set (patients with UM from Institut Curie; *N* = 560; left panel), validation series #1 (patients with UM from Institut Curie; *N* = 239; middle panel;) and validation series #2 (patients with UM from the ROMS cohort; *N* = 196; right panel). The ROC curves and AUC displayed correspond to the clinical model (in red) composed of patient’s age at diagnosis and largest basal diameter (LBD) of the tumor; the genetic model (in green) composed of genotypes of *IRF4* and *HERC2* loci coded as 0, 1, or 2 according to the number of risk alleles carried; and the combined model (in blue) that is the combination of the 2 previous models. The diagonal gray line corresponds to an AUC of 0.5, indicating randomness.

We then constructed 3 classifiers with the significantly associated variables using logistic regression and trained on the same data set: (1) a genetic classifier based on *HERC2* and *IRF4* genotypes, (2) a clinical classifier based on LBD and patient age, and (3) a mixed classifier combining genetic and clinical features (Table S10, available at https://www.ophthalmologyscience.org). Performance was evaluated by receiver operating characteristic area under the curve on 2 independent validation UM series (Fig 7). Interestingly, the genetic model predicted chromosome 3 status in the validation series with area under the curve of 0.62 (95% CI, 0.55–0.69) and 0.6 (95% CI, 0.53–0.67), respectively. The clinical classifier yielded similar performance, whereas the mixed classifier performed slightly better, although with overlapping CIs (Fig 7). Likelihood ratio tests showed that the mixed model was significantly better than the clinical (*P* < 0.001) and genetic (*P* < 0.001) models (Table S11, available at https://www.ophthalmologyscience.org).

**Figure 8:**
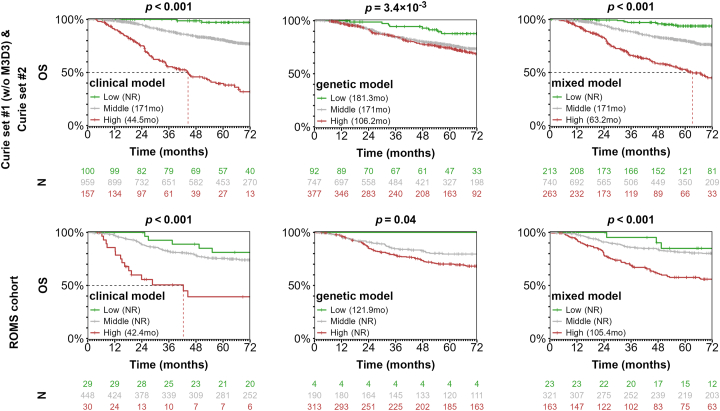
Kaplan-Meier (KM) analyses according to the predicted risk of the different models. KM plots associated with overall survival (OS) curves of patients with UM stratified by the risk predicted by the clinical model (left panel), the genetic model (middle panel), and the combined model (right panel). Each model was applied to 2 independent validation series: patients with UM from Institut Curie samples not used in model training (*N* = 1216; top panel) and the ROMS cohort (*N* = 507; lower panel). Median time in months is shown in parenthesis (NR = median not reached). Number of individuals for each KM analysis and for each prediction group is shown below the KM graph. *P* values were obtained from log-rank test. Censored individuals are noted with a vertical bar.

We then assessed the performance of our predictive models of being M3 on all UM cases without missing features and not included in the training series (Institut Curie: *N* = 1216 cases; ROMS cohort: *N* = 507 cases) using the OS as a surrogate of M3 UM. We classified each UM case into low (≤0.33), medium (>0.33 to ≤0.66), or high-risk (>0.66) according to the probability of being M3 obtained by each classifier. We then compared the OS of the 3 predicted groups for each classifier with Kaplan-Meier curves (Fig 8). Importantly, the genetic classifier had a significant predictive value on the outcome for the 2 validation UM series. However, the clinical and, to a lesser extent, the mixed classifiers performed better. The mild performance of the mixed classifier led us to suspect some redundancy between the clinical and the genetic classifiers. Indeed, both *IRF4* and *HERC2* genotypes were associated with tumor LBD and thickness but in opposite directions (Table S12, available at https://www.ophthalmologyscience.org). In summary, our results show that a classifier based on *HERC2* and *IRF4* SNPs is predictive of patient outcome but did not outperform current clinical prognostic markers.

## Discussion

On an extended series of UM cases, more than doubling the previously reported series,^16^ we confirmed the specific associations of *HERC2* and *IRF4* SNPs with M3 and D3 UM risk, respectively. Furthermore, we found that the *IRF4* risk genotype was associated with both specific D3 UM subtypes, namely *SF3B1-* and *EIF1AX-*mutated UMs. Individuals of European ancestry with light eye color are at a higher risk of developing UM.^10^^,^^11^ In our work, we showed blue eye pigmentation was specifically associated with a higher risk of M3 UM. This association was not found in the large series of patients with UM from The Netherlands using clinically registered eye colors.^28^ However, our analyses strongly support the risk of developing a D3 or M3 UM conferred by *IRF4* and *HERC2* SNPs, respectively, to be largely independent of their role in eye color determination. Actually, IRF4 and HERC2 have been implicated in different biological pathways besides their role in eye pigmentation, such as T cell development, immunity, and stemness.^29^, ^30^, ^31^, ^32^ Although we suspect that IRF4 and HERC2 may modulate the terminal differentiation of the melanocyte, along with their key roles in melanogenesis, the actual biological mechanism by which they modulate the risk of developing a D3 or M3 UM remains to be elucidated.

We then hypothesized that *HERC2* and *IRF4* genotypes would also be associated with UM survival given their respective associations with UM subtypes of opposite prognoses. Indeed, we observed improved PFS and OS in patients carrying the *IRF4* risk allele (associated with good prognosis) and, conversely, worse PFS and OS in patients carrying the *HERC2* risk allele (associated with poor prognosis). A conditional analysis confirmed that these associations with prognosis were mediated by chromosome status and were thus due to UM-related events. Interestingly, the recent publication by Gelmi et al^21^ from the group of M.J. Jager also showed the association of *HERC2* rs12913832-G with M3 status and UM survival but without identifying the opposite association with *IRF4* SNPs in this patient series from The Netherlands.

To evaluate the predictive power of *HERC2* and *IRF4* genotypes, we built a genetic classifier of chromosome 3 status and applied this classifier to predict outcomes in patients for whom tumor status was not available. Some limitations of our classifier were found. First, the genetic classifier did not perform as well in patients from the ROMS cohort (The Netherlands). The lower performance of the classifier can most likely be explained by the training set being mostly composed of French patients and the ROMS cohort set being mostly composed of Dutch patients. Indeed, the extreme variability of pigmentation gene allele frequencies in the different human populations (see Table 1) limits the generalizability of our results and implies the need for dedicated training of the tool for each population.^33^ This may also explain the differences observed between the French and Dutch patients for associations between patient risk genotype and outcome.^21^ Second, our genetic classifier did not outperform the clinical classifier, and the mixed classifier did not perform as well as expected. Interestingly, tumor LBD was the most important predictor in both clinical and mixed classifier, as expected from the American Joint Committee on Cancer staging criteria and recently confirmed in a very large series of patients with UM.^34^ These underwhelming results suggested some information redundancy between the genetic background and clinical features, such as higher LBD in M3 than in D3,^34^ as well as the association we found between *IRF4* and *HERC2* genotypes with tumor LBD and thickness. Finally, our classifiers were trained mainly in patients with large UMs treated by enucleation, which may have decreased the models’ performance on small UMs.

Altogether, our study demonstrates that inherited germline variants can inform on UM prognosis by predicting tumor subtype and survival. Although our current classifier does not yet surpass classic prognostic models, it establishes a critical proof-of-concept that the host genome carries valuable information about the biological trajectory of UM. This paves the way for the development of noninvasive genetic tools that could complement current prognostic assessments. Future studies in larger, multiancestry cohorts, coupled with integrative analyses of germline and tumor-specific features, will be essential to refine these tools and unlock their full clinical potential.
