# Supplementary material for: Genetic Background Predicts Uveal Melanoma Patients’ Outcomes
Source: Ophthalmol Sci. 2025 Oct 10;6(1):100972. doi: 10.1016/j.xops.2025.100972 (PMC12686906; doi:10.1016/j.xops.2025.100972)
Supplement: Figure S5 [file mmc1.pdf]

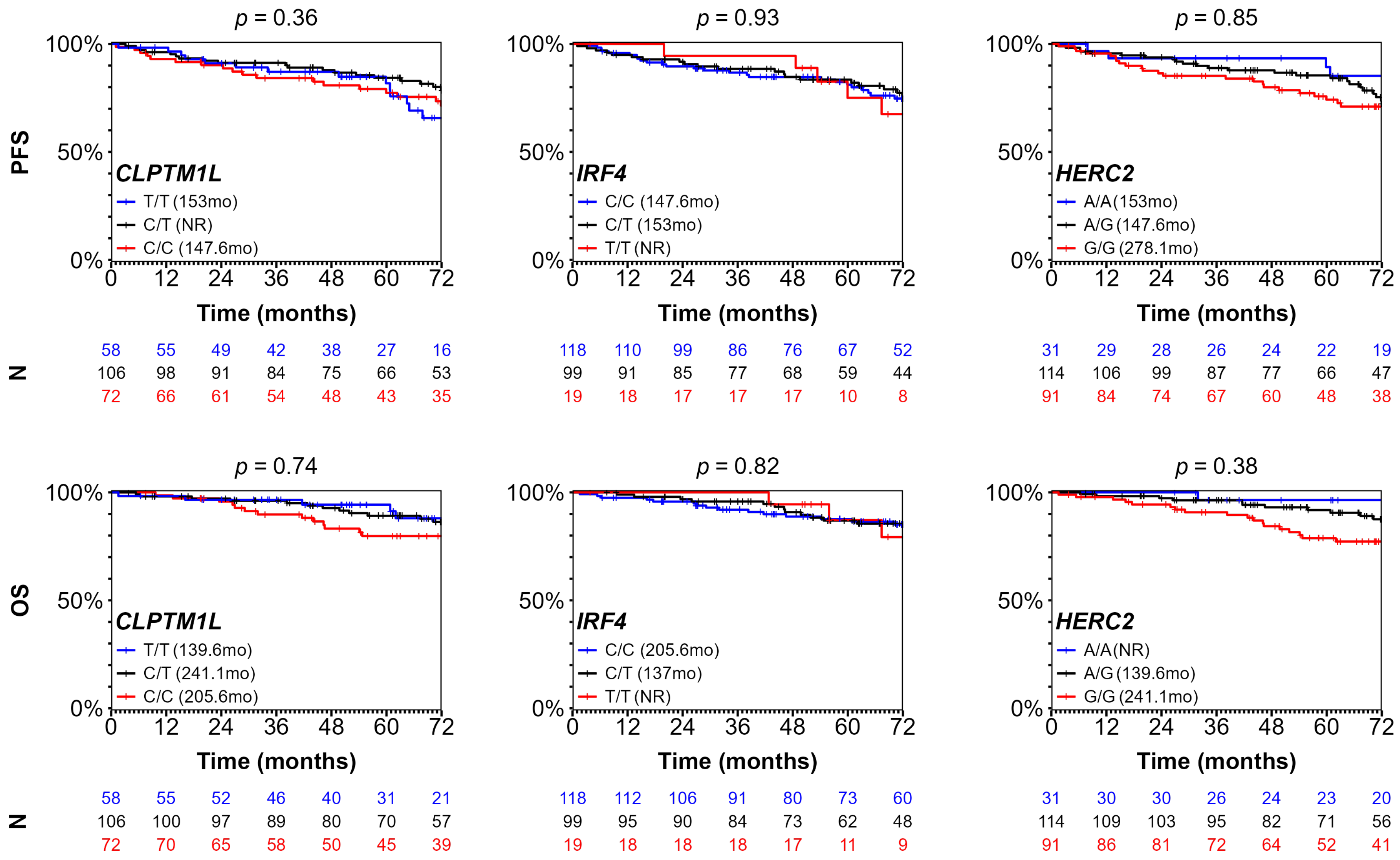

Figure S5. Kaplan Meier (KM) analyses of patients with disomy 3 (D3) uveal melanoma (UM) stratified by the 3 UM risk loci. KM plots of Progression Free Survival (PFS) and Overall Survival (OS) in 236 patients with D3 UM stratified by rs421284 (*CLPTM1L*; left panel); rs12203592 (*IRF4*; middle panel) and rs12913832 (*HERC2;* right panel). Median time in months is shown in parenthesis (NR = median Not Reached). *P*-value were obtained from log rank test with genotypes coded as 0, 1 or 2 according to the number of risk alleles carried. Number of individuals for each KM analysis and for all genotypes is shown below the KM graph. Censored individuals are noted with a vertical bar.
