# Supplementary material for: Genetic Background Predicts Uveal Melanoma Patients’ Outcomes
Source: Ophthalmol Sci. 2025 Oct 10;6(1):100972. doi: 10.1016/j.xops.2025.100972 (PMC12686906; doi:10.1016/j.xops.2025.100972)
Supplement: Supplementary Table 2 [file mmc2.pdf]

Table S2. Multivariate logistic regression on the chromosome 3 status.

Covariates	Features	N	*p* -value	OR§ (95% CI$)
Age at diagnosis		507	2.3e-03	1.02 (1.01 to 1.04)
Sex	Male	267		
	Female	240	0.37	1.18 (0.82 to 1.7)
Eye color	Brown	199		
	Green	50	0.15	0.63 (0.33 to 1.17)
	Blue	258	0.02	1.59 (1.09 to 2.34)

§: OR: odds-ratio
$: CI confidence interval
