# Supplementary material for: Genetic Background Predicts Uveal Melanoma Patients’ Outcomes
Source: Ophthalmol Sci. 2025 Oct 10;6(1):100972. doi: 10.1016/j.xops.2025.100972 (PMC12686906; doi:10.1016/j.xops.2025.100972)
Supplement: Supplementary Table 3 [file mmc3.pdf]

Table S3. Multivariate logistic regressions on the chromosome 3 status.

			CLPTM1L			
			With eye color		Without eye color	
**Covariates**	**Features**	**N**	***p*-value**	**OR[§] (95% CI[$])**	***p*-value**	**OR (95% CI)**
Risk locus		507	0.79	0.97 (0.75 to 1.25)	0.79	0.97 (0.75 to 1.24)
Age at diagnosis		507	2.5e-03	1.02 (1.01 to 1.04)	2.0e-03	1.02 (1.01 to 1.04)
Sex	Male	267				
	Female	240	0.39	1.18 (0.82 to 1.69)	0.38	1.17 (0.82 to 1.68)
Eye color	Brown	199			-	-
	Green	50	0.15	0.63 (0.33 to 1.18)	-	-
	Blue	258	0.02	1.6 (1.09 to 2.35)	-	-

			IRF4			
			With eye color		Without eye color	
**Covariates**	**Features**	**N**	***p*-value**	**OR (95% CI)**	***p*-value**	**OR (95% CI)**
Risk locus		507	< 0.001	0.45 (0.32 to 0.62)	< 0.001	0.44 (0.32 to 0.61)
Age at diagnosis		507	1.2e-03	1.02 (1.01 to 1.04)	< 0.001	1.02 (1.01 to 1.04)
Sex	Male	267				
	Female	240	0.42	1.16 (0.8 to 1.69)	0.41	1.17 (0.81 to 1.68)
Eye color	Brown	199			-	-
	Green	50	0.31	0.71 (0.37 to 1.36)	-	-
	Blue	258	0.01	1.63 (1.1 to 2.41)	-	-

			HERC2			
			With eye color		Without eye color	
**Covariates**	**Features**	**N**	***p*-value**	**OR (95% CI)**	***p*-value**	**OR (95% CI)**
Risk locus		507	1.8e-03	1.99 (1.3 to 3.08)	< 0.001	1.75 (1.33 to 2.32)
Age at diagnosis		507	3.7e-03	1.02 (1.01 to 1.04)	4.3e-03	1.02 (1.01 to 1.03)
Sex	Male	267				
	Female	240	0.4	1.17 (0.81 to 1.69)	0.45	1.15 (0.8 to 1.65)
Eye color	Brown	199			-	-
	Green	50	0.02	0.44 (0.22 to 0.87)	-	-
	Blue	258	0.4	0.77 (0.42 to 1.4)	-	-

§: OR: odds-ratio
$: CI confidence interval
