# Supplementary material for: Genetic Background Predicts Uveal Melanoma Patients’ Outcomes
Source: Ophthalmol Sci. 2025 Oct 10;6(1):100972. doi: 10.1016/j.xops.2025.100972 (PMC12686906; doi:10.1016/j.xops.2025.100972)
Supplement: Supplementary Table 4 [file mmc4.pdf]

Table S4. Multivariate logistic regressions on UM risk comparing *BAP1* -, *EIF1AX* - and *SF3B1* mutated UMs.

		CLPTM1L								
		**M3/*BAP1* mut**			***SF3B1* mut**			***EIF1AX* mut**		
**Covariates**	**Features**	**N**	***p* -value**	**OR[§] (95% CI[$])**	**N**	***p* -value**	**OR (95% CI)**	**N**	***p* -value**	**OR (95% CI)**
Risk locus		1212	1.4e-03	1.37 (1.13 to 1.66)	938	0.08	1.47 (0.95 to 2.29)	940	0.09	1.42 (0.94 to 2.14)
Age at diagnosis		1212	0.36	0.99 (0.98 to 1.01)	938	< 0.001	0.96 (0.93 to 0.98)	940	0.19	0.98 (0.96 to 1.01)
Sex	Male	817			681			697		
	Female	395	< 0.001	3.04 (2.33 to 3.98)	257	< 0.001	3.86 (2.15 to 7.02)	243	0.97	1.01 (0.51 to 1.89)

		IRF4								
		**M3/*BAP1* mut**			***SF3B1* mut**			***EIF1AX* mut**		
**Covariates**	**Features**	**N**	***p* -value**	**OR (95% CI)**	**N**	***p* -value**	**OR (95% CI)**	**N**	***p* -value**	**OR (95% CI)**
Risk locus		1212	0.26	1.16 (0.89 to 1.5)	938	< 0.001	2.55 (1.54 to 4.17)	940	< 0.001	2.35 (1.46 to 3.72)
Age at diagnosis		1212	0.3	0.99 (0.98 to 1.01)	938	< 0.001	0.96 (0.93 to 0.98)	940	0.13	0.98 (0.96 to 1.01)
Sex	Male	817			681			697		
	Female	395	< 0.001	3.03 (2.32 to 3.96)	257	< 0.001	3.85 (2.13 to 7.04)	243	0.84	1.07 (0.54 to 2.01)

		HERC2								
		**M3*/*BAP1* mut**			***SF3B1* mut**			***EIF1AX* mut**		
**Covariates**	**Features**	**N**	***p* -value**	**OR (95% CI)**	**N**	***p* -value**	**OR (95% CI)**	**N**	***p* -value**	**OR (95% CI)**
Risk locus		1212	< 0.001	2.03 (1.65 to 2.5)	938	0.4	1.2 (0.79 to 1.84)	940	0.08	1.46 (0.97 to 2.25)
Age at diagnosis		1212	0.21	0.99 (0.98 to 1)	938	< 0.001	0.96 (0.93 to 0.98)	940	0.18	0.98 (0.96 to 1.01)
Sex	Male	817			681			697		
	Female	395	< 0.001	2.95 (2.25 to 3.88)	257	< 0.001	3.89 (2.16 to 7.06)	243	0.98	1.01 (0.51 to 1.89)

§: OR: odds-ratio

$: CI confidence interval

*: M3: monosomy of chromosome 3
