# Supplementary material for: Genetic Background Predicts Uveal Melanoma Patients’ Outcomes
Source: Ophthalmol Sci. 2025 Oct 10;6(1):100972. doi: 10.1016/j.xops.2025.100972 (PMC12686906; doi:10.1016/j.xops.2025.100972)
Supplement: Supplementary Table 5 [file mmc5.pdf]

Table S5. Pairwise comparisons of survival times by log-rank test according to each risk SNP genotypes

Locus	Genotype 1	Genotype 2	PFS*	OS§
CLPTM1L rs421284	C/T	T/T	0.08	0.24
	C/C	T/T	0.08	0.24
	C/C	C/T	0.79	0.75
IRF4 rs12203592	C/T	C/C	< 0.001	2.2e-03
	T/T	C/C	0.02	0.02
	T/T	C/T	0.28	0.24
HERC2 rs12913832	A/G	A/A	0.13	0.16
	G/G	A/A	0.04	0.04
	G/G	A/G	0.13	0.16

*: PFS; progression free survival. P_value of the log-rank test.
§: OS; overall survival. P_value of the log-rank test.
