# Supplementary material for: Genetic Background Predicts Uveal Melanoma Patients’ Outcomes
Source: Ophthalmol Sci. 2025 Oct 10;6(1):100972. doi: 10.1016/j.xops.2025.100972 (PMC12686906; doi:10.1016/j.xops.2025.100972)
Supplement: Supplementary Table 6 [file mmc6.pdf]

Table S6. Univariate Cox proportional hazard model regressions on overall survival (OS) and progression free survival (PFS).

			OS		PFS	
**Covariates**	**Features**	**N**	**p-value**	**HR* (95% CI§)**	**p-value**	**HR (95% CI)**
CLPTM1L rs421284-C		1339	0.06	0.89 (0.79 to 1.01)	0.14	0.91 (0.8 to 1.03)
IRF4 rs12203592-T		1339	< 0.001	0.72 (0.61 to 0.84)	< 0.001	0.71 (0.61 to 0.84)
HERC2 rs12913832-G		1339	6.8e-03	1.19 (1.05 to 1.36)	7.7e-03	1.2 (1.05 to 1.38)
Sex	Male	655				
	Female	684	0.06	0.85 (0.72 to 1.01)	0.06	0.84 (0.7 to 1.01)
Age at diagnosis		1339	< 0.001	1.02 (1.02 to 1.03)	< 0.001	1.03 (1.02 to 1.04)
Tumor largest basal diameter		1339	< 0.001	1.21 (1.19 to 1.24)	< 0.001	1.18 (1.15 to 1.2)
Tumor thickness		1339	< 0.001	1.18 (1.15 to 1.2)	< 0.001	1.15 (1.12 to 1.18)
TNM	T1	282				
	T2	417	< 0.001	1.81 (1.3 to 2.54)	3.9e-03	1.68 (1.18 to 2.38)
	T3	451	< 0.001	3.73 (2.73 to 5.1)	< 0.001	2.97 (2.14 to 4.12)
	T4	189	< 0.001	9.47 (6.82 to 13.14)	< 0.001	6.81 (4.85 to 9.57)
Chromosome 3 status	Disomy 3	236				
	Monosomy 3	324	< 0.001	5.1 (3.86 to 6.73)	< 0.001	4.88 (3.58 to 6.65)

*: HR: Hazard-ratio

$: CI confidence interval
