# Supplementary material for: Genetic Background Predicts Uveal Melanoma Patients’ Outcomes
Source: Ophthalmol Sci. 2025 Oct 10;6(1):100972. doi: 10.1016/j.xops.2025.100972 (PMC12686906; doi:10.1016/j.xops.2025.100972)
Supplement: Supplementary Table 7 [file mmc7.pdf]

Table S7. Multivariate Cox proportional hazard model regression on overall survival (OS) and progression free survival (PFS)

Covariates	Features	N	OS		PFS	
			p-value	HR* (95% CI[§])	p-value	HR (95% CI)
CLPTM1L rs421284-C		1339	0.07	0.89 (0.79 to 1.01)	0.16	0.91 (0.8 to 1.04)
IRF4 rs12203592-T		1339	< 0.001	0.77 (0.66 to 0.9)	5.0e-03	0.79 (0.67 to 0.93)
HERC2 rs12913832-G		1339	0.05	1.14 (1 to 1.3)	0.03	1.18 (1.02 to 1.35)
Sex	Male	655				
	Female	684	0.16	0.88 (0.74 to 1.05)	0.13	0.87 (0.72 to 1.04)
Age at diagnosis		1339	< 0.001	1.02 (1.02 to 1.03)	< 0.001	1.04 (1.03 to 1.04)
Tumor largest basal diameter		1339	< 0.001	1.19 (1.16 to 1.22)	< 0.001	1.16 (1.13 to 1.19)
Tumor thickness		1339	0.1	1.03 (0.99 to 1.06)	0.11	1.03 (0.99 to 1.07)

*: HR: Hazard-ratio

§: CI confidence interval
