# Supplementary material for: Genetic Background Predicts Uveal Melanoma Patients’ Outcomes
Source: Ophthalmol Sci. 2025 Oct 10;6(1):100972. doi: 10.1016/j.xops.2025.100972 (PMC12686906; doi:10.1016/j.xops.2025.100972)
Supplement: Supplementary Table 8 [file mmc8.pdf]

Table S8. Multivariate Cox proportional hazard model regression including the chromosome 3 status.

Covariates	Features	N	OS		PFS	
			**p-value**	**HR* (95% CI§)**	**p-value**	**HR (95% CI)**
CLPTM1L rs421284-C		560	0.35	0.93 (0.8 to 1.08)	0.63	0.96 (0.81 to 1.14)
IRF4 rs12203592-T		560	0.8	1.03 (0.84 to 1.26)	0.92	1.01 (0.81 to 1.27)
HERC2 rs12913832-G		560	0.46	1.07 (0.9 to 1.27)	0.28	1.11 (0.92 to 1.35)
Sex	Male	294				
	Female	266	0.08	0.82 (0.65 to 1.03)	0.12	0.82 (0.64 to 1.05)
Age at diagnosis		560	0.02	1.01 (1 to 1.02)	< 0.001	1.02 (1.01 to 1.03)
Tumor largest basal diameter		560	< 0.001	1.14 (1.1 to 1.18)	< 0.001	1.11 (1.06 to 1.15)
Tumor thickness		560	0.86	1 (0.95 to 1.04)	0.75	1.01 (0.96 to 1.06)
Chromosome 3 status	Disomy 3	236				
	Monosomy 3	324	< 0.001	4.44 (3.32 to 5.95)	< 0.001	4.15 (2.99 to 5.74)

*: HR: Hazard-ratio

§: CI confidence interval
