# Supplementary material for: Genetic Background Predicts Uveal Melanoma Patients’ Outcomes
Source: Ophthalmol Sci. 2025 Oct 10;6(1):100972. doi: 10.1016/j.xops.2025.100972 (PMC12686906; doi:10.1016/j.xops.2025.100972)
Supplement: Supplementary Table 9 [file mmc9.pdf]

Table S9. Univariate logistic regression on the chromosome 3 status.

Covariates	Features	N	p-value	OR§ (95%CI$)
IRF4 rs12203592-T		560	< 0.001	0.46 (0.34 to 0.62)
HERC2 rs12913832-G		560	< 0.001	1.75 (1.35 to 2.27)
Sex	Male	294		
	Female	266	0.06	1.39 (0.99 to 1.95)
Age at diagnosis		560	1.7e-03	1.02 (1.01 to 1.03)
Tumor largest basal diameter		560	< 0.001	1.11 (1.06 to 1.17)
Tumor thickness		560	0.42	1.02 (0.97 to 1.08)

§: OR: odds-ratio

$: CI confidence interval
