# Supplementary material for: Genetic Background Predicts Uveal Melanoma Patients’ Outcomes
Source: Ophthalmol Sci. 2025 Oct 10;6(1):100972. doi: 10.1016/j.xops.2025.100972 (PMC12686906; doi:10.1016/j.xops.2025.100972)
Supplement: Supplementary Table 11 [file mmc11.pdf]

Table S10. Fitted model coefficients.

Covariates	N	Genetic model		Clinical model		Mixed model	
		p-value	OR§ (95% CI$)	p-value	OR (95% CI)	p-value	OR (95% CI)
IRF4 rs12203592-T	560	< 0.001	0.47 (0.35 to 0.64)	-	-	< 0.001	0.48 (0.35 to 0.65)
HERC2 rs12913832-G	560	< 0.001	1.71 (1.31 to 2.23)	-	-	< 0.001	1.61 (1.23 to 2.11)
Age at diagnosis	560	-	-	5.0e-03	1.02 (1.01 to 1.03)	7.0e-03	1.02 (1.01 to 1.03)
Tumor largest basal diameter	560	-	-	< 0.001	1.11 (1.06 to 1.17)	< 0.001	1.1 (1.04 to 1.15)

§: OR: odds-ratio

$: CI confidence interval
