# Supplementary material for: Genetic Background Predicts Uveal Melanoma Patients’ Outcomes
Source: Ophthalmol Sci. 2025 Oct 10;6(1):100972. doi: 10.1016/j.xops.2025.100972 (PMC12686906; doi:10.1016/j.xops.2025.100972)
Supplement: Supplementary Table 12 [file mmc12.pdf]

Table S11. Nested fitted models comparisons by asymptotic likelihood-ratio tests

Genetic versus mixed models

Model	Degrees of freedom	log-likelihood	likelihood ratio Chi2 statistic	*p*-value
Genetic	3	-359.88	-	-
Mixed	5	-348.68	22.39	< 0.001

Clinical versus mixed models

Model	Degrees of freedom	log-likelihood	likelihood ratio Chi2 statistic	*p*-value
Clinical	3	-367.1	-	-
Mixed	5	-348.68	36.83	< 0.001
