# Supplementary material for: Genetic Background Predicts Uveal Melanoma Patients’ Outcomes
Source: Ophthalmol Sci. 2025 Oct 10;6(1):100972. doi: 10.1016/j.xops.2025.100972 (PMC12686906; doi:10.1016/j.xops.2025.100972)
Supplement: Supplementary Table 13 [file mmc13.pdf]

Table S12. Characteristics of 1339 patients with UM from Institut Curie set #1 according to each risk SNP genotype

	CLPTM1L			**p-value**	**IRF4**			**p-value**	**HERC2**			**p-value**
**Genotype**	**T/T**	**C/T**	**C/C**		**C/C**	**C/T**	**T/T**		**A/A**	**A/G**	**G/G**	
**N**	**303**	**664**	**372**		**776**	**501**	**62**		**154**	**504**	**681**	
**Tumor characteristics**												
Chromosome 3 status#				0.5				<0.001				<0.001
Disomy 3	58 (43%)	106 (40%)	72 (46%)		118 (34%)	99 (52%)	19 (73%)		31 (55%)	114 (50%)	91 (33%)	
Monosomy 3	76 (57%)	162 (60%)	86 (54%)		226 (66%)	91 (48%)	7 (27%)		25 (45%)	113 (50%)	186 (67%)	
Undetermined	169	396	214		432	311	36		98	277	404	
Tumor largest basal diameter§&	13.2 (4.1)	12.9 (4.2)	13.3 (4.2)	0.3	13.4 (4.2)	12.7 (4.1)	12.3 (4.1)	0.007	12.3 (4.0)	13.1 (4.2)	13.2 (4.2)	0.049
Tumor thickness&	7.0 (3.6)	6.6 (3.4)	6.9 (3.6)	0.2	7.1 (3.6)	6.5 (3.4)	6.4 (3.4)	0.008	6.7 (3.4)	7.1 (3.5)	6.6 (3.6)	0.018
**Individual characteristics**												
Sex#				0.2				0.3				0.7
Male	138 (46%)	321 (48%)	196 (53%)		394 (51%)	231 (46%)	30 (48%)		75 (49%)	254 (50%)	326 (48%)	
Female	165 (54%)	343 (52%)	176 (47%)		382 (49%)	270 (54%)	32 (52%)		79 (51%)	250 (50%)	355 (52%)	
Age at diagnosis&	63 (14)	62 (14)	61 (13)	0.088	62 (14)	62 (13)	62 (15)	>0.9	60 (14)	61 (14)	63 (13)	0.2
Eye color*#				0.6				<0.001				<0.001
Brown	107 (39%)	228 (37%)	118 (34%)		274 (38%)	164 (35%)	15 (27%)		127 (89%)	307 (66%)	19 (3.0%)	
Green	29 (11%)	65 (10%)	44 (13%)		64 (8.8%)	59 (13%)	15 (27%)		10 (7.0%)	82 (18%)	46 (7.2%)	
Blue	139 (51%)	330 (53%)	189 (54%)		391 (54%)	241 (52%)	26 (46%)		6 (4.2%)	79 (17%)	573 (90%)	
Unknown	28	41	21		47	37	6		11	36	43	

n (%); Mean (SD)
*: eye color is clinically registered
§: LBD largest basal diameter as measured by B-mode ultrasonography
#: Pearson's Chi-squared test
&: Kruskal-Wallis rank sum test
